# Supplementary material for: Physical Interpretation of Diffractive Optical Networks for High‐Dimensional Vortex Mode Sorting
Source: Adv Sci (Weinh). 2025 Oct 16;13(1):e14100. doi: 10.1002/advs.202514100 (PMC12767018; doi:10.1002/advs.202514100)
Supplement: Supplementary file 1 — Supporting Information [file ADVS-13-e14100-s001.pdf]

Supplementary Materials for

Physical Interpretation of Diffractive Optical Networks for High-Dimensional Vortex Mode Sorting

Ruitao Wu,† Juncheng Fang,† Rui Pan, Rongyi Lin, Kaiyuan Li, Ting Lei,* Luping Du,* and Xiaocong Yuan*

Nanophotonics Research Centre, Institute of Microscale Optoelectronics & State Key Laboratory of Radio Frequency Heterogeneous Integration, Shenzhen University, Shenzhen 518060, China.

* Corresponding authors: leiting@szu.edu.cn, lpdu@szu.edu.cn, xcyuan@szu.edu.cn

Supplementary Note 1: Laguerre-Gaussian modes

Here, we briefly give the necessary background for the modes we used in the main text, the Laguerre-Gaussian (LG) modes[1]. Before proceeding, one should note that there is a fundamentally strict definition of "modes" and "beams", since "beams" are propagating three-dimensional light fields and "modes" are transversal (or longitudinal) two-dimensional fields. However, the difference is not critical in the context of what we discussed in this paper, as we mainly focused on scenarios under paraxial conditions. The readers can consult other literature[2] for in-depth discussions. We note that the terms "LG modes" and "high-dimensional vortex modes" are used interchangeably in this paper.

Mathematically, the LG beams are solutions of the Helmholtz equation in circular cylindrical coordinates in free space. Its field distribution can be described using[1]:

$$E(r,\theta,z) = \frac{A_0}{W(z)}\left(\frac{\sqrt{2}\,r}{W(z)}\right)^{|l|} \exp\left(-\frac{r^2}{W(z)^2}\right) L_p^{|l|}\left(\frac{2r^2}{W(z)^2}\right) \exp\left(ikz - \frac{ikr^2}{2R(z)} - il\theta + i\psi_G\right), \qquad (S1)$$

where A_0 is the normalized constant, $W(z) = W_0\sqrt{1+(z/z_0)^2}$ is the beam radius, with $W_0 = \sqrt{\lambda z_0/\pi}$. The wavelength is denoted by λ, which is related to the wavevector $k = 2\pi/\lambda$. z_0 quantifies the Rayleigh range. $L_p^{|l|}$ gives the generalized Laguerre polynomials, where l and p are the azimuthal index and radial index, respectively. $R(z) = z[1+(z_0/z)^2]$ is the radius of curvature for the wavefront, which needs to be taken into account when designing diffractive networks. $\psi_G = (2p + |l| + 1)\tan^{-1}(z/z_0)$ is the Gouy phase, a factor in which the sorting functionality has also been investigated[3].

Supplementary Note 2: Retrieving log-polar transformation for vortex mode sorting

In the first section of the paper, we have trained a two-layer diffractive network that performs the vortex mode sorting task. Such a problem has been widely investigated since 2010, with the first analytical diffractive mask solution demonstrated in the pioneering work by G. C. Berkhout et al[4]. Here, we provided the necessary theoretical background of this transformation, with details regarding how to retrieve the exact transformation function in our trained systems.

The idea of the "log-polar sorter" is to first perform a log-polar transformation using two diffractive phase masks. One of the masks will perform as an "unwrapper" while the other one works as the "correction" to achieve an afocal optical system[5]. This will turn the azimuthal phase gradients of vortex modes into a linear phase gradient along the Cartesian coordinate (say the x-axis). The unwrapper phase on mask 1 can be described as:

$$\phi_{12}(x_1,y_1) = \frac{k}{d_{12}}\left\{a\left[x_1\, arctan\left(\frac{x_1}{y_1}\right) - y_1\, ln\left(\sqrt{\frac{x_1^2+y_1^2}{b}}\right) + y_1\right] - \frac{x_1^2+y_1^2}{2}\right\}, \qquad (S2)$$

where the parameter a is the input beam size and b is the targeted beam size in the output. d_{12} is the distance between the two masks.

The correction phase on mask 2 is given as:

$$\phi_{21}(x_2,y_2) = -\frac{k}{d_{12}}\left\{ab\left[\exp\left(-\frac{y_2}{a}\right) cos\left(\frac{x_2}{a}\right)\right] - \frac{x_2^2+y_2^2}{2}\right\}, \qquad (S3)$$

Once the coordinate transformation is performed, a lens is used later to perform the focusing. The output fields will be Gaussian-like spots with displacements along the x-axis since the lens performs a Fourier transform for the transformed tilted plane-wave-like modes. Besides, a phase term performing focusing (at

output distance f), which is given by $\phi_f(x_2, y_2) = -\frac{k}{2f}(x_2^2 + y_2^2)$, can be added to the correction mask. Therefore, a minimum of two masks is required. In this paper, we used $f = 50\,mm$ in both numerical calculations and experiments for demonstration purposes.

In practice, we note that for LG beam and vortex beam inputs, an additional phase arises during the free space propagation (with distance d_0) and needs to be compensated. As described in Equation (S1), this additional phase term is given by $\exp\left(-\frac{ikr^2}{2R(d_0)}\right)$, associated with the laser mode curvatures upon propagation. A phase conjugation term $\exp[i\phi_c(x_1, y_1)] = \exp\left(\frac{ikr^2}{2R(d_0)}\right)$ can be applied to the first diffractive layer to eliminate this distortion. Such a compensation process is not needed if the input plane overlaps with the first mask.

Considering all the terms discussed above, we can identify that the appropriate function for mask 1 is given as:

$$\phi_1 = \phi_c + \phi_{12} = \frac{kr_1^2}{2R(d_0)} + \frac{k}{d_{12}}\left\{a\left[x_1\,arctan\left(\frac{x_1}{y_1}\right) - y_1\,ln\left(\sqrt{\frac{x_1^2 + y_1^2}{b}}\right) + y_1\right] - \frac{x_1^2 + y_1^2}{2}\right\}. \quad (S4)$$

For mask 2, we obtained:

$$\phi_2 = \phi_{21} + \phi_f = -\frac{k}{d_{12}}ab\left[\exp\left(-\frac{y_2}{a}\right)cos\left(\frac{x_2}{a}\right)\right] + k\left(\frac{1}{d_{12}} - \frac{1}{f}\right)\frac{x_2^2 + y_2^2}{2}. \quad (S5)$$

For Figure 2, we have obtained the trained masks that perform the one-dimensional vortex mode sorting task. The output sorted modes used for initializing our design are distributed in a way that is very similar to the analytical log-polar vortex mode sorter, meaning they are distributed along the x-axis and are symmetric along the y-axis in terms of their topological charges. As we mentioned in the main text, even though the masks are initially flat, the trained network has exhibited phase patterns that are akin to the log-polar vortex mode sorter. Therefore, we performed the fitting of both trained layers with unknown parameters a, b, and f, using Equations (S4) and (S5), respectively. We note that only the ROI regimes (indicated by red dotted lines) are used for fitting. Their regimes are identified numerically by transmitting the LG modes (training set) to the trained masks, as these are the only regions where the designed phases are actually "working".

The fields shown in the "transformed" column in Figure 2d in the main text are calculated using the term in Equation (S4) after removing the focusing phase term. The field similarities between the trained network and our analytical model have proved that the trained network has produced physical processes that are easily understood by the designers. Such an idea could be further expanded to high-dimensional tasks with diffractive systems with increasing complexity, as we demonstrated in the next section.

Supplementary Note 3: Physical transformation processes for 2D LG mode sorting

We proceed to provide the analysis of physical transformation processes for LG mode sorting into two-dimensional laser spot arrays. As we discussed in the main text, there are three diffractive layers within the trained network, which potentially involve two transformations. The processes within the trained masks can be retrieved by analyzing the phase patterns within the system. We assigned the phase distributions within these three masks with ϕ_1, ϕ_2 and ϕ_3, respectively.

First of all, considering our compensation procedure for the propagation upon the first masks, the first masks can be split into two terms $\phi_1 = \phi_c + \phi_{12}$, the compensation term ϕ_c, as well as the transformation term ϕ_{12}. Similarly, we have $\phi_2 = \phi_{21} + \phi_{23}$ and $\phi_3 = \phi_{32} + \phi_f$. ϕ_{12} and ϕ_{21}, ϕ_{23} and ϕ_{32}, are two phase distribution pairs that perform the physical transformation processes. ϕ_f is the focusing term that is

responsible for the final sorting procedure. Figure S4 summarizes the detailed physical meaning of each term and how they are identified, as we will discuss in detail below.

The knowledge on the first transformation can be obtained by checking the distribution of $\phi_{12} = \phi_1 - \phi_c$ using the trained ϕ_1 and prior knowledge of ϕ_c. One could see that ϕ_{12} performs the circular-sector transformation, which can be further validated by propagating the input modes to the first mask and checking the field distributions. This transformation has gained great attention for OAM sorting, multiplication, and division[6]. This process is similar to the log-polar transformation we mentioned previously, but with a distinct phase mask pair. It transforms the phase gradient along the azimuthal axis from 2pi to a limited range such that it can be transformed into spatially varying spots with a lens phase. In other words, it is mainly responsible for sorting the azimuthal indices. Typically, the unwrapper phase on mask 1 is given by:

$$\phi_{12}(r_1, \theta_1) = \frac{k}{d_{12}} \left\{ a_1 b_1 \left(\frac{r_1}{b_1}\right)^{1-\frac{1}{n}} \cdot \frac{cos\left[\left(1-\frac{1}{n}\right)\theta_1 - \varphi_0\right]}{1-\frac{1}{n}} - \frac{r_1^2}{2} \right\}, \quad (S6)$$

with the polar coordinate r_1, θ_1. a_1 and b_1 are the scaling parameters. φ_0 stands for the azimuthal shift. n represents the ratio of the transformation.

The corresponding phase corrector in mask 2 is given by:

$$\phi_{21}(r_2, \theta_2) = \frac{k}{d_{12}} \left\{ a_1 b_1 \left(\frac{r_2}{b_1}\right)^{1-n} \cdot \frac{cos[(1-n)\theta_2 + n\varphi_0]}{1-n} - \frac{r_2^2}{2} \right\}. \quad (S7)$$

These two masks work synergistically to perform the circular-sector transformation. r_2, θ_2 also denote the polar coordinates.

Similar to the previous section, we can confirm the functionality of mask 1 by performing the fitting using functions that are derivatives of Equation (S6). We note that in practice, there is another y-axis reposition term in the mask to ensure the diffractive light energy is concentric with the input beam for the second plane (right before the second mask). Therefore, a phase shift term with a displacement factor y_0 is added in both masks to ensure the right transformation. With the aforementioned propagation phase compensation term, the exact function for mask 1 can be given as:

$$\phi_1 = \phi_c + \phi_{12} = \frac{kr_1^2}{2R(d_0)} + \frac{k}{d_{12}} \left\{ a_1 b_1 \left(\frac{r_1}{b_1}\right)^{1-\frac{1}{n}} \cdot \frac{cos\left[\left(1-\frac{1}{n}\right)\theta_1 - \varphi_0\right]}{1-\frac{1}{n}} - \frac{r_1^2}{2} + c_1 \frac{y_0^2}{2} \right\}. \quad (S8)$$

The number of variables used for the fitting is five, which includes $a_1, b_1, c_1, n,$ and y_0. c_1 denotes the length of displacements. φ_0, other the other hand, solely depends on the location of the fields due to our design. Once the parameters within ϕ_{12} are retrieved, we can calculate the corresponding phase correction term by adapting the analytical solution in Equation (S7):

$$\phi_{21} = \frac{k}{d_{12}} \left\{ a_1 b_1 \left(\frac{r_2}{b_1}\right)^{1-n} \cdot \frac{cos[(1-n)\theta_2 + n\varphi_0]}{1-n} - \frac{r_2^2}{2} - c_1 \frac{y_0^2}{2} \right\}, \quad (S9)$$

which finalizes our extraction on the first transformation process.

Next, we focus on extracting the phase pair for the second transformation. This process can be understood by obtaining ϕ_{23} and ϕ_{32}, respectively. Alternatively, we can calculate ϕ_2 and ϕ_3, as we demonstrate next. We note that this transformation is rather complicated, since it represents a beam reshaper that turns the input sector fields into relatively uniform fields with phase variations along the y-axis. These phase variations are key to sorting the radial indices p along the designed direction. The sorting effect along the x-axis is similar to the log-polar transformation except that now the focusing phase term is along a sector trajectory, with a curved lens phase in the last layer. It’s noteworthy that such a process might be interpreted as the inverse

process of multiple beam splitting in the Fresnel regime, except that the energy in each diffractive order is not uniform[7].

While there are no known rigorous analytical solutions for sorting the radial indices of LG modes, in our study, we impose the iterative optimization algorithm to numerically quantify the second transformation. The method we used is adapted from the approach that was initially proposed for designing the fan-out elements[8]. Briefly, we now consider a 2-layer structure by removing the known mask 1 from the 3-layer system. Since we have knowledge of both the original output fields (laser spot arrays) and the updated input fields (obtained by propagating each input mode through the original mask 1), it is possible to apply optimization algorithms to simultaneously calculate numerical solutions for both ϕ_2 and ϕ_3. Our approach is based on the gradient descent, with a target function evaluated by the similarities between all the target output fields and the optimized output fields defined for the optimization process $F = \sum_{i=1}^{n} \left| E_{optimized} \cdot conj(E_{target}) \right|^2$. The initial condition of the first mask is given by $\phi_2 = \phi_{21}$, while ϕ_3 starts from the random phase distribution. Once ϕ_2 and ϕ_3 are obtained, we can calculate ϕ_{23} using the relation $\phi_{23} = \phi_2 - \phi_{21}$, as shown in Figure 3b. ϕ_{21} is already known following Equation (S9). We note that this iterative optimization algorithm does not produce results that are significantly different than the wavefront matching approaches. An interesting observation is that, in our training in Figure 3, we have not succeeded in producing "swapped" solutions by manipulating the initial surface conditions. In other words, the circular-sector transformation always appears before the complex diffraction term for p indices. This particular solution is rather stable, as demonstrated in Figure S3. While other solutions might be theoretically possible (more appropriately said, they are not theoretically forbidden), the current solution we acquired seems to outperform the others and eventually survives after the multiple iterations of the algorithm. We noted that this observation also appears in networks with an increasing number of masks as well, meaning the solutions are rather stable.

Strictly speaking, since the second transformation is non-conformal, there is no perfect phase compensation mask exists (ϕ_{32}). However, for a finite number of p values, an approximate solution can be obtained to correct the phase distortion to a certain degree. Once ϕ_{23} is retrieved, an approximate solution of ϕ_{32} can be further calculated from the knowledge of ϕ_{23}. This is done by setting up a new uniform plane wave input (with a finite aperture to avoid edge effect) for a new two-layer system, which consists of only two masks with a separation distance d_{23}. The first mask is assigned with ϕ_{23}. The correction phase ϕ_{32} can be estimated by taking the phase conjugation of the field, as it is designed for correcting the phase distortion ϕ_{23}. Finally, the lensing phase term ϕ_f can be further evaluated by $\phi_f = \phi_3 - \phi_{32}$. In our case, the ϕ_f term we obtained is akin to a spherical lens distribution (Figure S4).

In summary, in this section, we have identified the functionalities within a three-layer diffractive network trained for LG mode sorting. Figure S4 summarizes all the physical meanings of the three masks and their relations. In particular, we have successfully demonstrated the retrieval of these two transformation processes. These two transformation processes are independent of each other, as they are designed to sort two indices, the radial and azimuthal factors, respectively, along two orthogonal axes. Besides, this independence is potentially boosted by the distinct physical meanings of these two indices.

Supplementary Note 4: Physical transformation division within complex networks

In Figure 4, we have illustrated how the physical transformation evolves within a complex network with an increasing number of masks. Here, we present the evolution of the LG beam intensity profile after it propagates through each mask, at four different propagation distances, respectively. To minimize redundancy, we will only show three mode index groups as examples, which include $LG_{2,0}$, $LG_{0,2}$, and $LG_{-1,2}$, respectively. Also, only cases of $N \geq 4$ is visualized since there is no satisfied solution for $N = 2$, while $N = 3$ is discussed extensively in Figure 3. We note that we used the term "transformer" to describe the element that performs the corresponding coordinate transformation.

Interestingly, for LG mode sorting purposes, the input field always goes through a coordinate transformation process first, then experiences a beam shaping process to compress the elongated beam. A repositioning

process is then involved to move the beam to the desired output location, with a final lens phase to focus it. These processes might be performed together by one mask or achieved by two cascaded masks. $LG_{2,0}$ is illustrated in Figure S6 to exemplify the OAM sorting process, while $LG_{0,2}$ serves as the example in Figure S7 for p index sorting along the y-axis. An example of simultaneous sorting of both indices is given in Figure S8.

Supplementary Note 5: Extrapolation of diffractive networks under system variations

The knowledge of physics behind a DN introduces many new opportunities for understanding and designing new DNs. For diffractive optical networks, it has been demonstrated that it is possible to use the trained networks under certain conditions[7]. However, the condition for the reported extrapolation method has been quite limited, as it requires simultaneous variation of other experimental conditions. Other typical extrapolation procedure requires retraining or modification of the model[9]. In this work, we have extended the discussion and demonstrated the extrapolation of DNs under system variations. Main results are shown in Figure 5. Here we present the detailed theoretical derivation and extrapolation procedure in the following.

We start by considering a network with layer number N illustrated in Figure S9. Each diffractive mask is labelled by sequence: 1, 2, …, N, with corresponding coordinates (x_1, y_1), (x_2, y_2),… (x_N, y_N). Specifically, the input plane is defined as plane 0 of coordinates (x_0, y_0) and the output plane is numbered by N+1 with (x_{N+1}, y_{N+1}). The distances between each adjacent mask pair are given by $d_0, d_1, d_2, \dots d_N$, labeled by the plane before. The pretrained masks are characterized by their phase distribution $\phi_N(x_N, y_N)$. Such a system can be modelled by a transfer matrix S, with the following equation:

$$S = H_N S_N H_{N-1} \cdots H_1 S_1 H_0, \qquad (S10)$$

where H_i denotes the transfer matrix for distance d_i, S_i represents the matrix for the ith mask with ϕ_i. Following our example on the 2- and 3-layer network, the phase for each mask can be classified according to its index (i is a positive integer taken from 1 and N):

$$\phi_i(x_i, y_i) = \begin{cases} \phi_c + \phi_{12}, & i = 1 \\ \phi_{i,i-1} + \phi_{i,i+1}, & i \in [2, N-1]. \\ \phi_{N,N-1} + \phi_f, & i = N \end{cases} \qquad (S11)$$

As we mentioned in previous examples, ϕ_c denotes the propagation phase that is associated with the curvature of the beam acquired, while ϕ_f represents the lens phase that appears when the task is designed for sorting. The intermediate terms, $\phi_{i,i+1}$ and $\phi_{i+1,i}$, perform the corresponding physical processes within the network and ensure the afocal condition. Theoretically, the transformation term is defined using the stationary phase approximation[5], following the conformal mapping of (x_i, y_i) to (x_{i+1}, y_{i+1}):

$$\frac{\partial \phi_{i,i+1}(x_i, y_i)}{\partial x_i} = \frac{k}{d_i}(x_{i+1} - x_i), \quad \frac{\partial \phi_{i,i+1}(x_i, y_i)}{\partial y_i} = \frac{k}{d_i}(y_{i+1} - y_i), \qquad (S12)$$

and the corresponding phase correction term:

$$\frac{\partial \phi_{i+1,i}(x_{i+1}, y_{i+1})}{\partial x_{i+1}} = \frac{k}{d_i}(x_i - x_{i+1}), \quad \frac{\partial \phi_{i+1,i}(x_{i+1}, y_{i+1})}{\partial y_{i+1}} = \frac{k}{d_i}(y_i - y_{i+1}). \qquad (S13)$$

While $\phi_{i,i+1}$ and $\phi_{i+1,i}$ are associated with each other through Equations (S12) and (S13), it is possible, but not straightforward, to directly and accurately evaluate $\phi_{i+1,i}$ from $\phi_{i,i+1}$ (or vice versa). Only a few analytical solutions, including the log-polar and circular-sector transformation, have been reported. The difficulties of solving this problem are further enhanced if the transformation is not strictly conformal.

However, given that $\phi_{i,i+1}$ and $\phi_{i+1,i}$ constitute an afocal system, meaning that $\phi_{i+1,i}$ is applied for correcting the phase distortion of input modes induced by $\phi_{i,i+1}$. Therefore, a straightforward approach is to consider a new two-layer network (separated by d_i) with $\phi_{i,i+1}$ and $\phi_{i+1,i}$, with plane-wave-like input modes for each layer. In this case, $\phi_{i+1,i}$ can be estimated by taking the conjugate of the phase distribution for modes right before the mask, since the output modes should take a uniform phase distribution.

Following this approach, we are able to evaluate all the phase terms within $\phi_i(x_i, y_i)$. Once all phase terms are known, we would be able to perform the extrapolation. Considering the new system parameters to be: wavevector k', and corresponding new distances $d_0', d_1', d_2', \dots d_N'$, we can obtain the extrapolated (denoted by the prime symbol) masks to be:

$$\phi_i'(x_i, y_i) = \begin{cases} \phi_c' + \dfrac{k'}{k}\dfrac{d_1}{d_1'}\phi_{12}, & i = 1 \\ \dfrac{k'}{k}\left[\dfrac{d_{i-1}}{d_{i-1}'}\phi_{i,i-1} + \dfrac{d_i}{d_i'}\phi_{i,i+1}\right], & i \in [2, N-1] \\ \dfrac{k'}{k}\left[\dfrac{d_{N-1}}{d_{N-1}'}\phi_{N,N-1} + \dfrac{d_N}{d_N'}\phi_f\right], & i = N \end{cases}. \tag{S14}$$

We would like to emphasize that, in Equation (S14), ϕ_c and ϕ_c' are both pre-knowledge of the system and can both be eliminated by enforcing $d_0 = d_0' = 0$ when designing the system. Alternatively, it can be considered as an extrapolation process with a new system with different input distances. We noted that they do not meet the same scaling equation, meaning $\phi_c' \neq \frac{k'}{k}\frac{d_0}{d_0'}\phi_c$, since the dependence of d and λ on the radius of curvature of the beam R is nonlinear.

For a typical MPLC system, the intermediate distances are identical. Assuming that only inter-plane distances and the input wavelength are scaling ($d_0' = d_0$ and $d_N' = d_N$), we can significantly simplify Equation (S14) to:

$$\phi_i'(x_i, y_i) = \begin{cases} \phi_c' + m\phi_{12}, & i = 1 \\ m\phi_i, & i \in [2, N-1] \\ m\phi_{N,N-1} + m_k\phi_f, & i = N \end{cases}, \tag{S15}$$

where $m = m_k m_d$ is the scaling factor, with $m_k = \frac{k'}{k} = \frac{\lambda}{\lambda'}$ representing the wavelength scaling, and $m_d = \frac{d_i}{d_i'}(i > 0)$ denoting the scaling on inter-plane distances. Equation (S15) describes the model we applied for the examples shown in Figure 5 in the main text.

We would like to emphasize that such extrapolation is general and does not require knowledge of explicit transformation. The accuracy of this approach solely depends on the conformal approximation[5]. For the 2-layer system (log-polar vortex mode sorter), such a condition is strictly guaranteed. Therefore, a theoretically perfect performance can be achieved after extrapolation. However, for the 3-layer system (or even more masks), the extrapolation will lead to degraded systems. Nevertheless, our system still outperforms the pre-trained masks. With the increased masks, such an approximation might be further inaccurate, since the numerical errors during the calculation will accumulate, leading to reduced performance.

Lastly, we would like to discuss the possibility of changing the mask orders once all the terms are obtained. Following Equation (S10), one might expect that the operations within each layer can be swapped. However, this can not be easily achieved using our approach since the ROI regions among the masks can be very different. Outside the ROI for each mask, the retrieved masks are not sufficiently accurate. Therefore, the performance of the system consisting of swapped phase masks can be significantly degraded.

Supplementary Figures

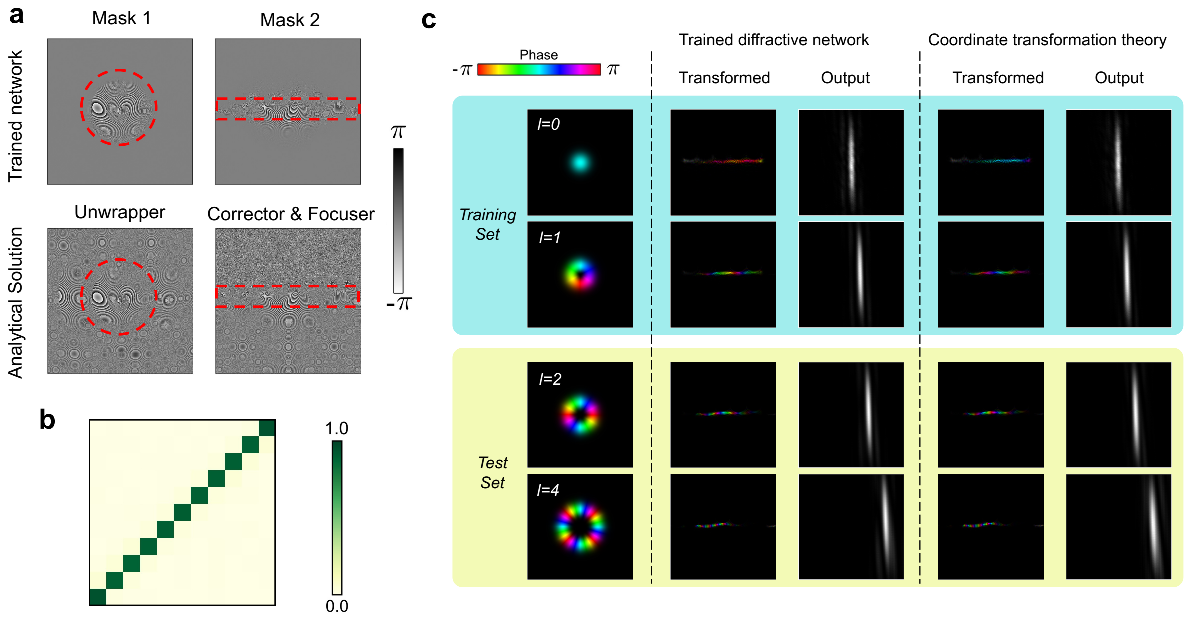

Figure S1. Physical interpretation of diffractive networks for a vortex mode sorting: re-discovery of the spiral transformation approach

The separation efficiency of a vortex mode sorter based on log-polar transformation can be improved by increasing the corresponding phase gradients for the transformed beams. An elegant way to achieve this is to apply the spiral transformation[10]. In this transformation, an OAM mode of topological charge l is transformed into a rectangular-shaped field with a phase gradient of nl through spiral mapping, where n represents the ratio of enhancement. The sorting process is done by incorporating a lens phase into the second mask, which converts the rectangular-shaped field into focused spots. In this work, similar to the log-polar sorter example (Figure 2), we have trained a network that can reproduce the physical process of the spiral sorter for OAM modes. All system parameters are identical to the log-polar sorter in the main text, except that the output spots are modified to be Gaussian-like modes by focusing the rectangular shape fields. (a) Comparison of a spiral sorter obtained from the analytical model (top) and the trained network (bottom). The trained mask pairs resemble the spiral transformation, while one mask acts as the unwrapper and the other one serves as the corrector and the focuser. Red dotted lines indicate the region of interest, where optical power is focused in each layer. (b) The detection efficiency of this trained network is evaluated to be 89%, which can be further improved by increasing the corresponding phase gradient ratio. (c) Verification of the transformation process for both the spiral mode sorter and the trained network. The images show the phase of numerical model light fields (colorful images) and the intensity (grayscale images). We note that the intensity images are magnified by 2 times with respect to the field images. The sorted spots are elongated in the y-axis as transformed rectangular fields are compressed in the second layer of the system.

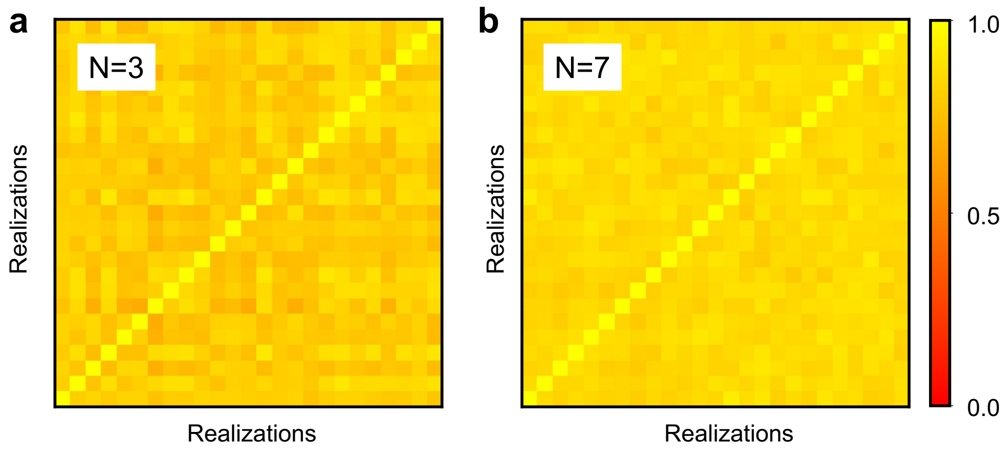

Figure S2. Stability investigation of trained network solutions

To test the stability of our training process, we have performed simulations with 25 different realizations and calculated the cross-correlation coefficients among them. We note that for different realizations, the training set (mode number of 28) and test set (mode number of 5) are randomly chosen from the 33 LG modes, while all other parameters remain the same. The tests are performed for systems with two different mask numbers, (a) N=3 and (b) N=7, respectively. All training starts from flat surfaces. It's evident that the output mask solutions are stable, regardless of the choice of the modes used for training.

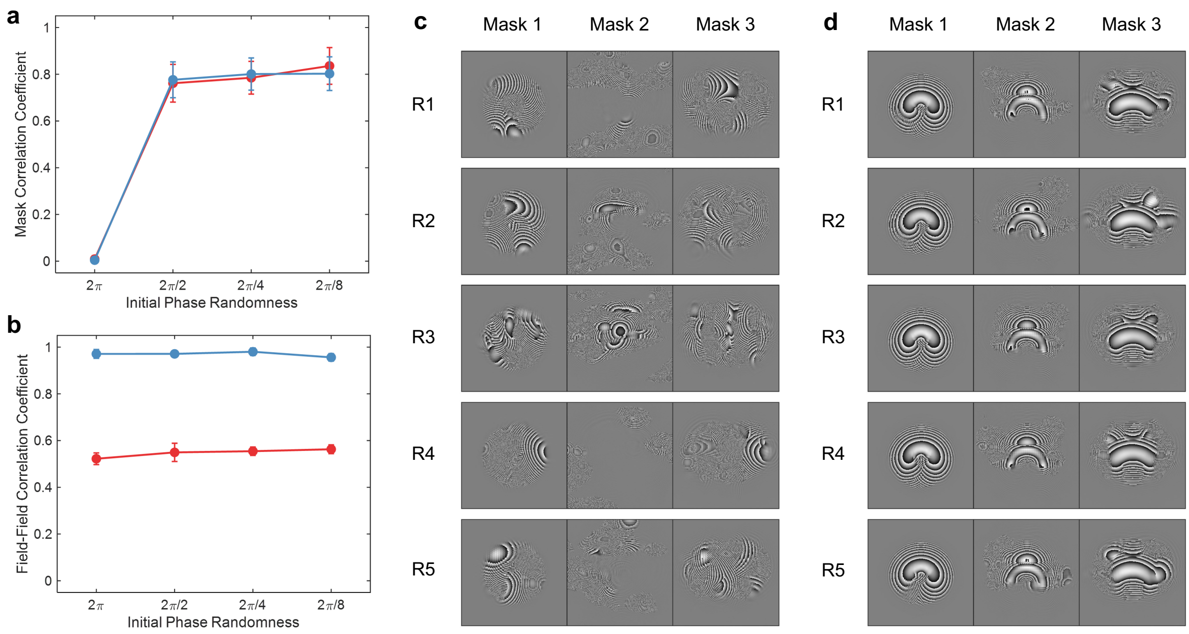

Figure S3. Effect of initial phase conditions on trained outcome

The stability of the training results is tested with different initial phase conditions assigned for the masks. The system under investigation is the three-layer system discussed in the main test. Simulations are performed for initial masks with uniform phase distribution for a range from 0 to 2pi/M, where M is a positive integer. (a) The mask solutions are stable except for the strongest randomness (phase from 0 to 2pi), as high mask correlation coefficients are obtained. (b) The output field-field correlation coefficients are given, indicating that all systems will lead to almost identical system performance. In both (a) and (b), the blue line indicates the correlation coefficients with the masks trained with flat surfaces, while the red line indicates the correlation coefficients among five different realizations. The error bar indicates the standard deviations among the five realizations and three masks. (c) Illustration of the mask solutions obtained from the five realizations under the strongest randomness (phase from 0 to 2pi). (d) Illustration of the mask solutions obtained from the five realizations under partial randomness (phase from 0 to pi). These results indicate that, assigning a completely initially random phase distribution to the masks before training will lead to unstable solutions and masks with vague physical meanings. However, the system still shows indistinguishable performances compared to the stable solutions obtained under other conditions (partially random phase and flat surfaces).

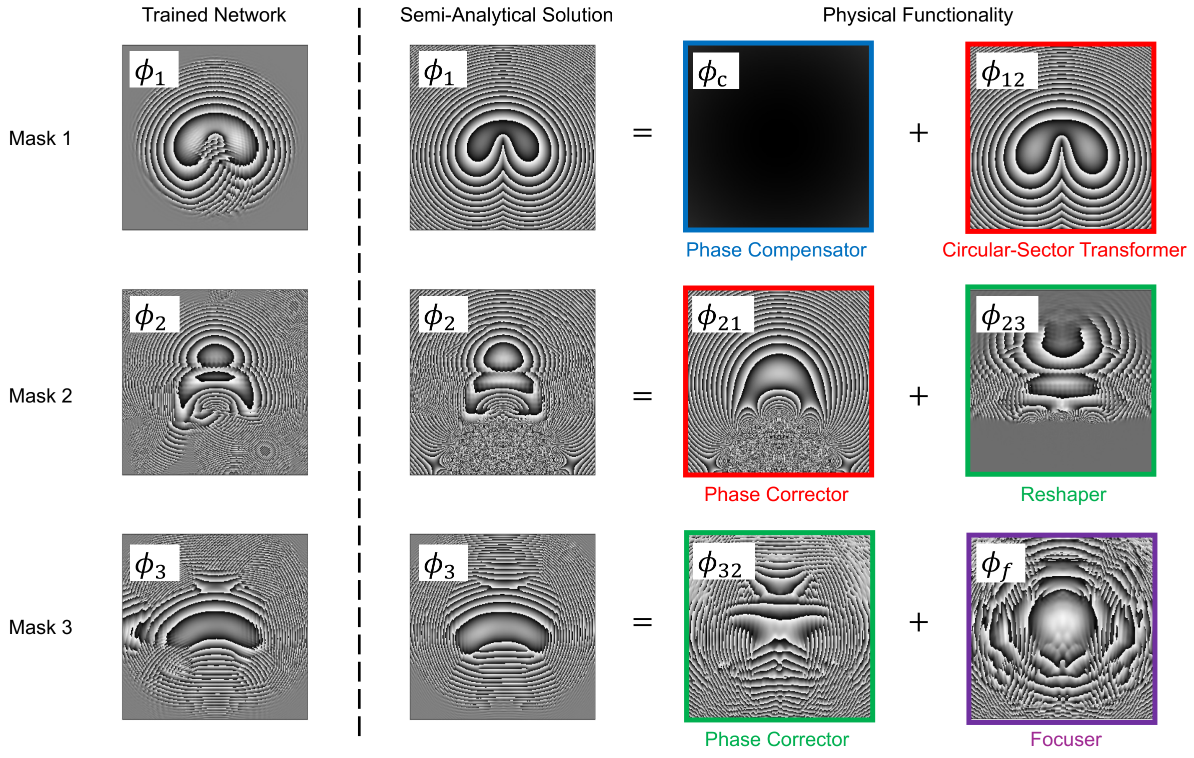

Figure S4. Identifying the physical meaning of each mask within the triple-layer network

Illustration of physical functionalities on all layers within the three-layer network trained for LG mode sorting. Each mask contains two distinct phase components. Mask 1 consists of the phase compensator ϕ_c and the circular-sector transformer ϕ_{12}. The phase correction for ϕ_{12} is ϕ_{23} within ϕ_2. ϕ_{12} and ϕ_{23} perform the first physical transformation: the circular sector transformation. Another term in ϕ_2 is the ϕ_{23}, which performs complex mode reshaping, mainly responsible for the p index sorting. Such a process is the second transformation, and it is compensated by ϕ_{32} in the third mask. ϕ_3 also contains the focusing term ϕ_f for performing the mode sorting. See Supplementary Note 3 for details on retrieving these terms.

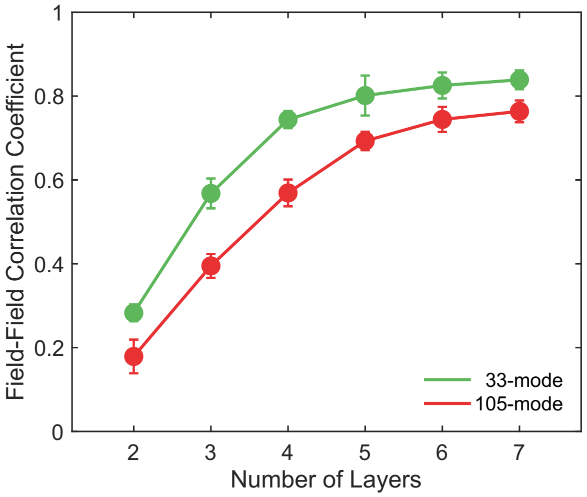

Figure S5. Performance analysis of multi-layer diffractive networks trained for LG mode classification: effect of mode set numbers

In Figure 4, we have conducted the analysis on the performance of a trained network (as a high-dimensional vortex mode sorter) as a function of the number of layers. That network under investigation is trained with 33 LG modes (5 modes are picked as the test set and the rest are used as the training set), spanning from l from -5 and 5, p from 0 to 2, respectively. Here, we further investigated the performance of another network trained with an increased number of LG modes, while all other parameters are kept the same. Figure S5 (red curve) plots the performance of such a network trained with 105 LG modes in total (l from -11 to 11, p from 0 to 4). The 33-mode case (green curve) is shown for comparison. Interestingly, while the phase distribution in each layer for the 105-mode case is not identical to the 33-mode case (as the beam size profiles have increased), the functionality of each mask remains the same as its peers in Figure 4, regardless of the number of layers. The cut-off for the overfitting and underfitting regions remains identical, meaning the change of system performance becomes negligible at $N \geqslant 6$ and $N \leqslant 2$. This observation is coherent with our conclusion in the main text that there is a correlation between the field-field correlation coefficient and the redundancy of the solutions. The system performance, however, is degraded with an increasing number of modes, as the value of field correlation for the 105-mode case is lower than that of the 33-mode. Such a decrease can be expected since the number of pixels per mode is reduced while the total available pixels remain identical.

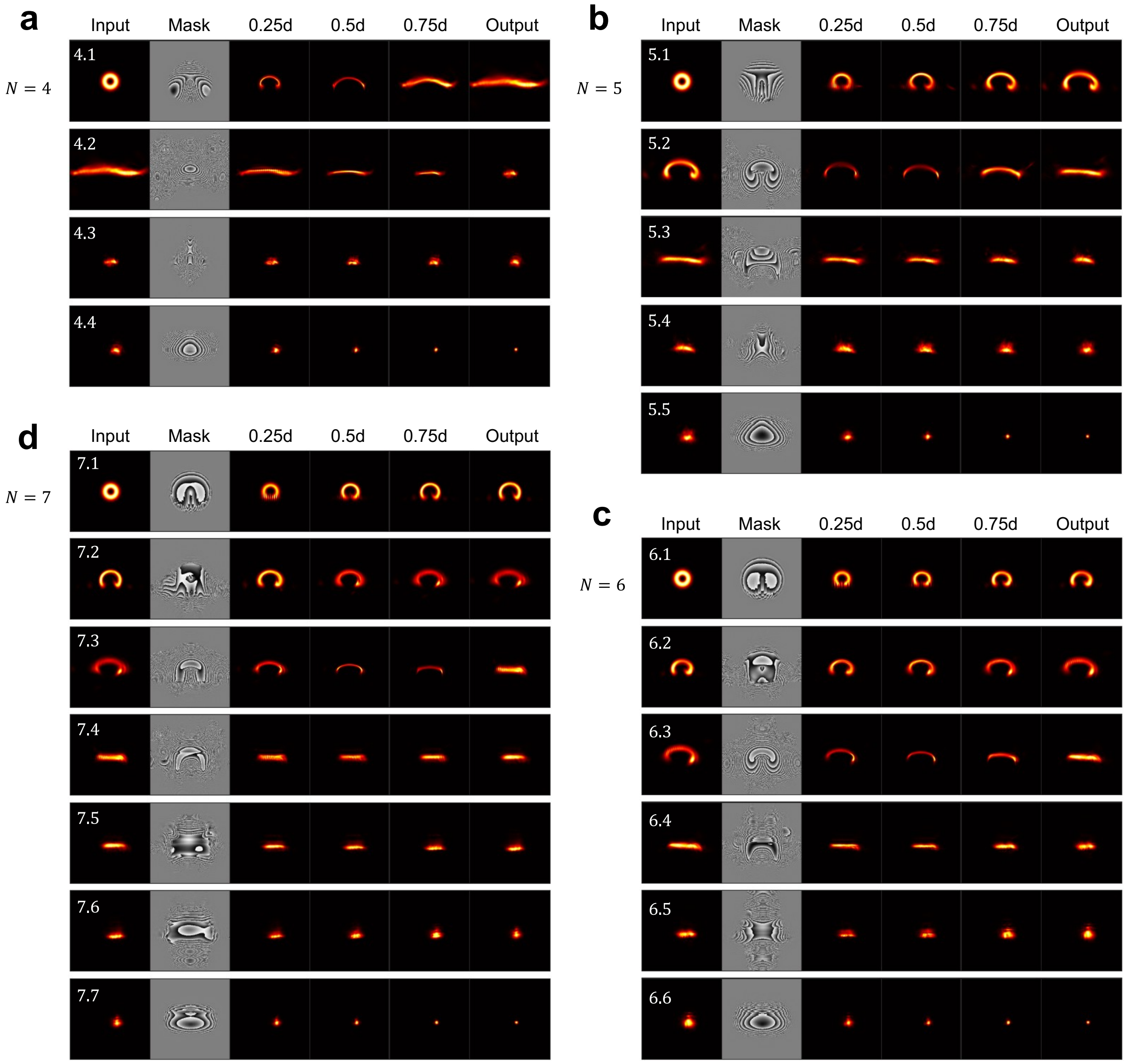

Figure S6. $LG_{2,0}$ intensity evolution for DNs of different numbers of diffractive layers

Details of the physical transformation process are visualized by comparing these DNs. One can clearly see the coordinate transformation processes involved, such as log-polar transformation (a-4.1), circular-sector transformation (b-5.1), or sector-cartesian transformation (b-5.2, c-6.2, d-7.3). The physical transformation process is also observed, for instance, the compressor in c-6.4 is split into d-7.4 and d-7.5.

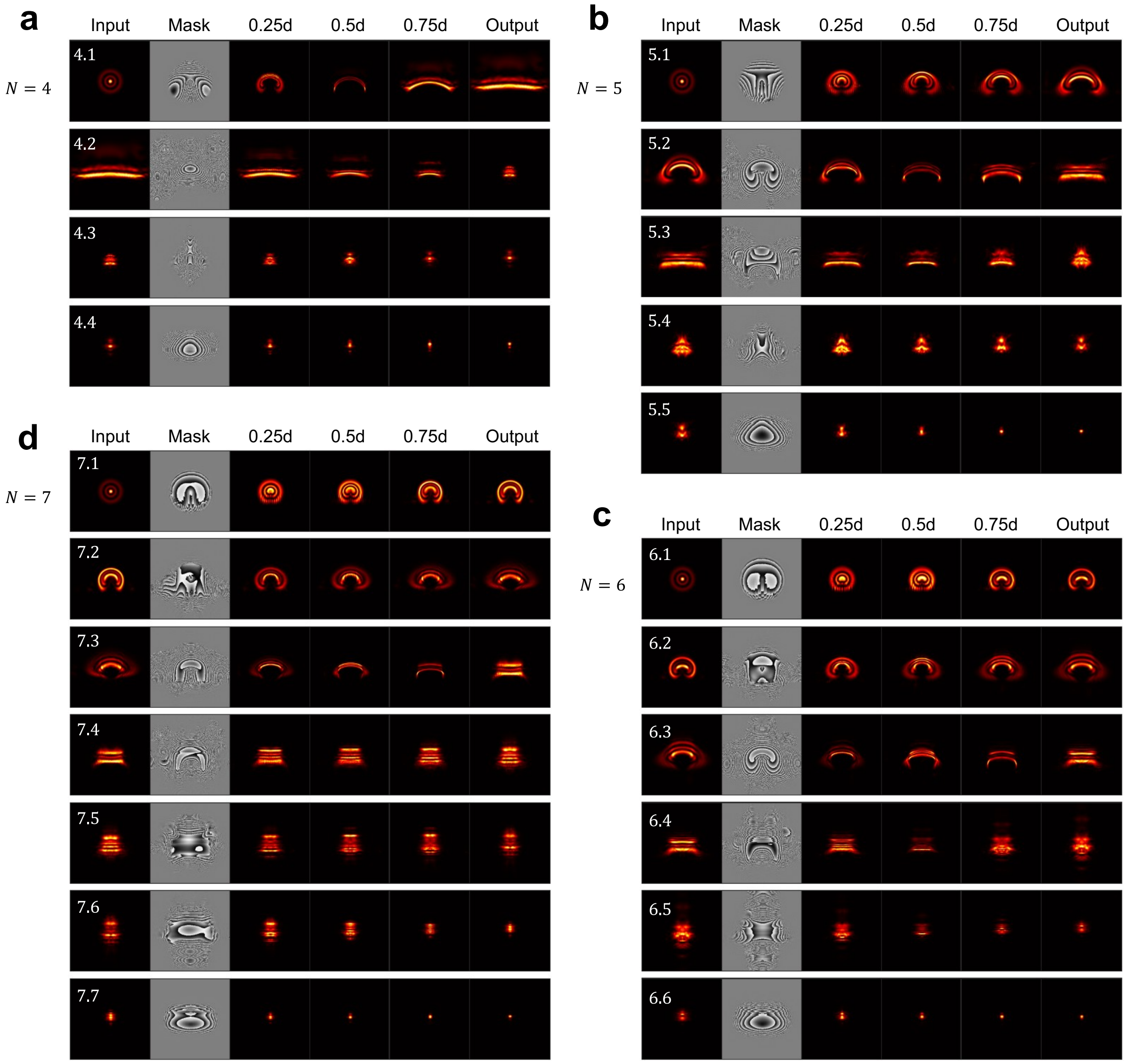

Figure S7. $LG_{0,2}$ intensity evolution for DNs of different numbers of diffractive layers

Same as Figure S6, but now emphasizing the sorting of the p indices.

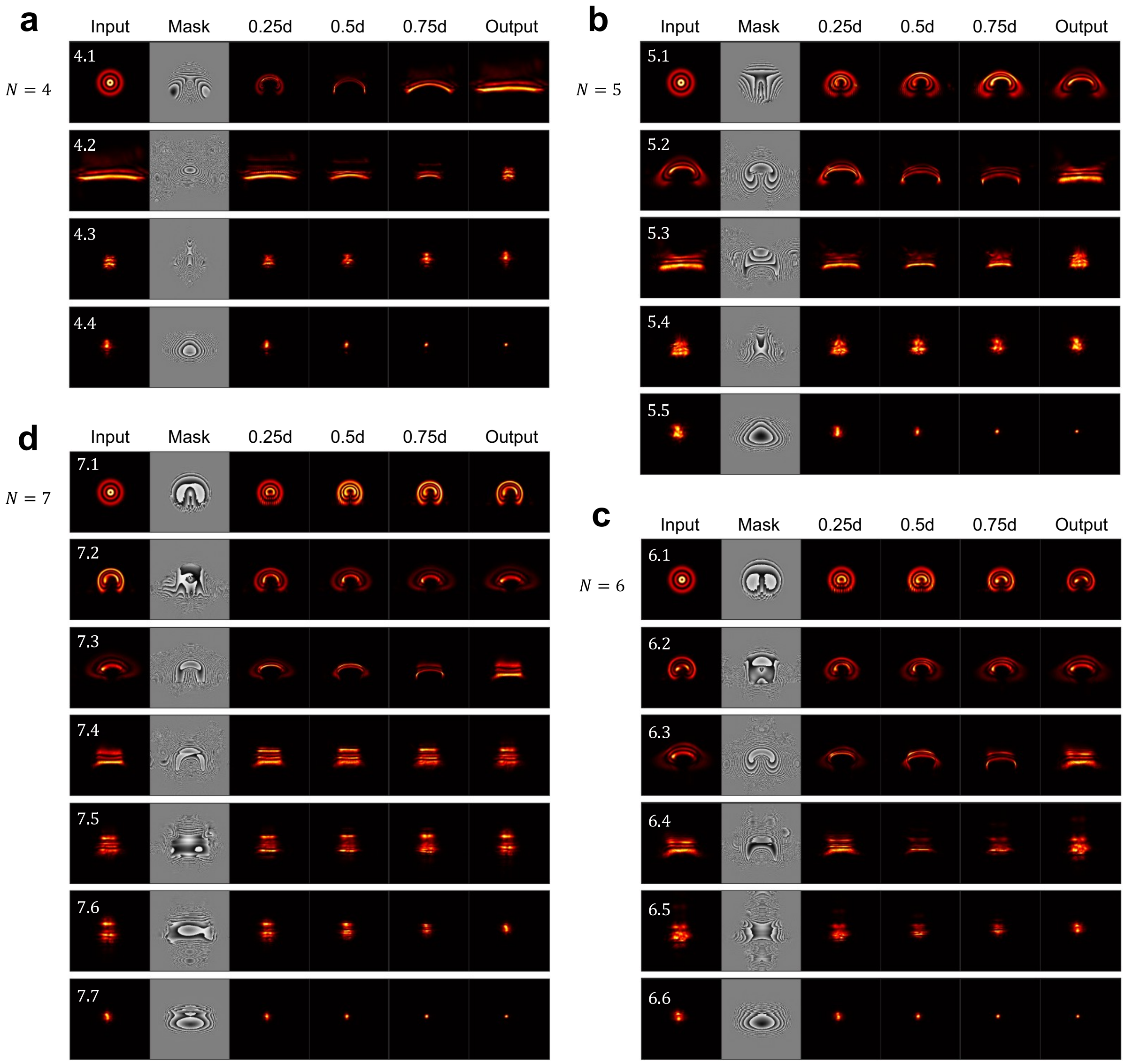

Figure S8. $LG_{-1,2}$ intensity evolution for DNs of different numbers of diffractive layers

Same as Figure S6, but now emphasizing the simultaneous sorting of both l and p indices.

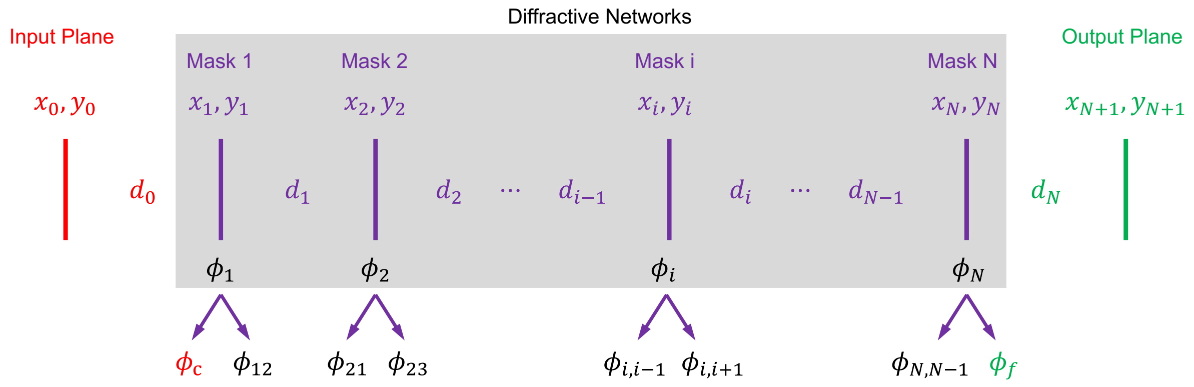

Figure S9. Illustration of functionalities within sequential multiple diffractive networks

Each mask can be split into two terms according to its functionalities, as one performs the phase correction while the other performs the new transformation, except for the first and last masks. The first mask has an additional term for compensating the phase curvature carried by the propagating beam, while the last mask requires a focusing term that performs the sorting task.

References

[1] L. Allen, M. W. Beijersbergen, R. Spreeuw, J. Woerdman, "Orbital angular momentum of light and the transformation of Laguerre-Gaussian laser modes", *Physical Review A* **1992**, *45* (11), 8185.

[2] D. A. Miller, "Waves, modes, communications, and optics: a tutorial", *Advances in Optics and Photonics* **2019**, *11* (3), 679-825.

[3] Y. Zhou, M. Mirhosseini, D. Fu, et al., "Sorting photons by radial quantum number", *Physical Review Letters* **2017**, *119* (26), 263602.

[4] G. C. Berkhout, M. P. Lavery, J. Courtial, M. W. Beijersbergen, M. J. Padgett, "Efficient sorting of orbital angular momentum states of light", *Physical Review Letters* **2010**, *105* (15), 153601.

[5] W. Hossack, A. Darling, A. Dahdouh, "Coordinate transformations with multiple computer-generated optical elements", *Journal of Modern Optics* **1987**, *34* (9), 1235-1250.

[6] G. Ruffato, M. Massari, F. Romanato, "Multiplication and division of the orbital angular momentum of light with diffractive transformation optics", *Light: Science & Applications* **2019**, *8* (1), 113.

[7] N. K. Fontaine, R. Ryf, H. Chen, et al., "Laguerre-Gaussian mode sorter", *Nature Communications* **2019**, *10* (1), 1865.

[8] D. Prongué, H.-P. Herzig, R. Dändliker, M. T. Gale, "Optimized kinoform structures for highly efficient fan-out elements", *Applied Optics* **1992**, *31* (26), 5706-5711.

[9] A. R. Kong, T. Lei, J. C. Fang, et al., "Achromatic broadband multi - layer diffraction mode multiplexing", *Laser & Photonics Reviews* **2023**, *17* (6), 2200845.

[10] Y. Wen, I. Chremmos, Y. Chen, et al., "Spiral transformation for high-resolution and efficient sorting of optical vortex modes", *Physical Review Letters* **2018**, *120* (19), 193904.
